# Supplementary material for: SNAREs define targeting specificity of trafficking vesicles by combinatorial interaction with tethering factors
Source: Nat Commun. 2019 Apr 8;10:1608. doi: 10.1038/s41467-019-09617-9 (PMC6453939; doi:10.1038/s41467-019-09617-9)
Supplement: Supplementary file 1 — Supplementary Information [file 41467_2019_9617_MOESM1_ESM.pdf]

Supplementary Information

SNAREs define targeting specificity of trafficking vesicles by combinatorial interaction with tethering factors

Seiichi Koike and Reinhard Jahn

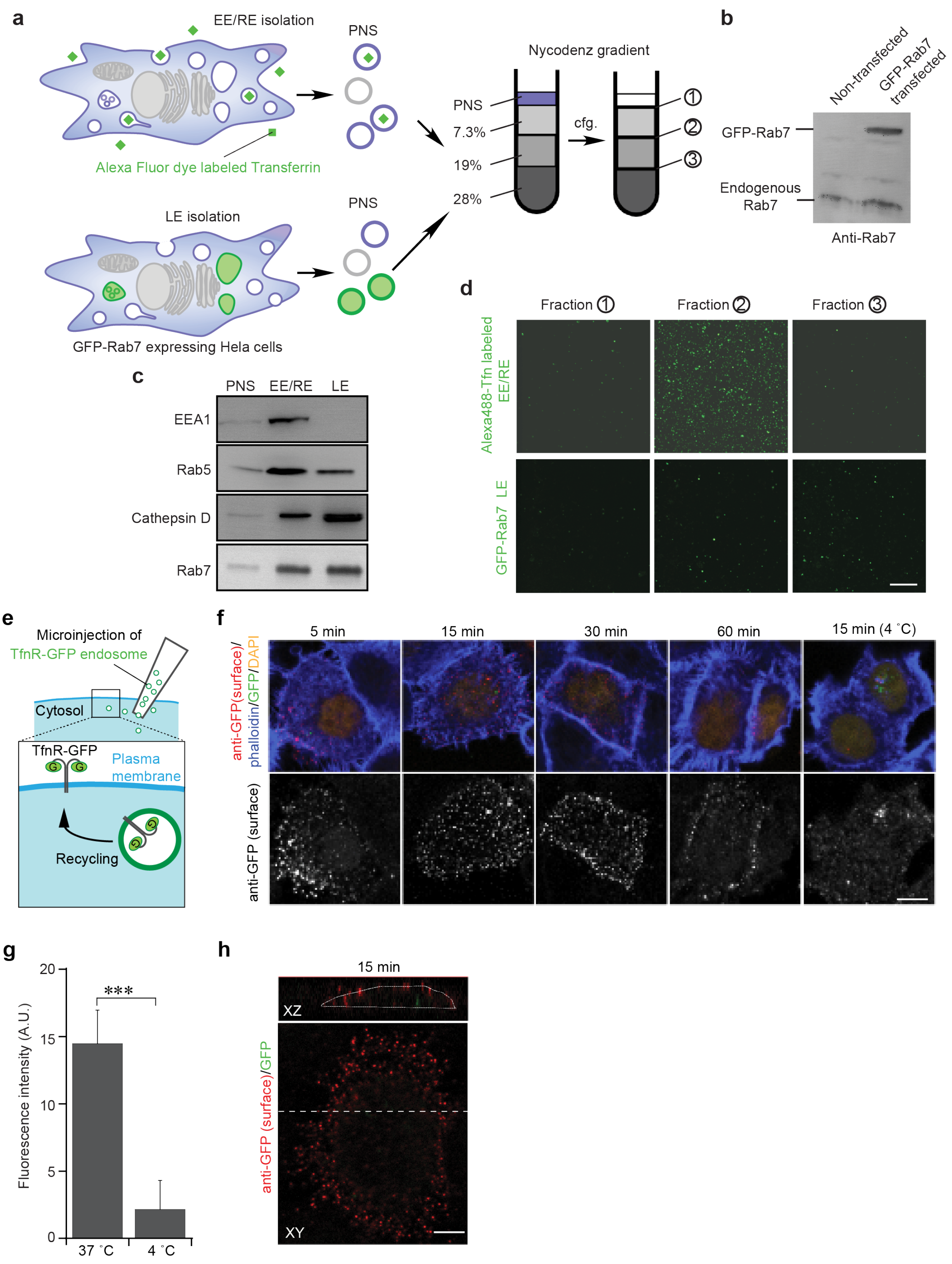

Supplementary Figure 1. Isolation of functional endosomes

a, Schematic overview over the procedure for isolating early/recycling endosomes (EE/RE) and late endosomes, respectively from HeLa cells. To label early endosomes, cells were incubated for 5 min with Alexa Fluor 488-labeled transferrin (50 µg/ml), which was internalized by

endocytosis and is commonly used as marker for early/recycling endosomes. Late endosomes (LE) were isolated from HeLa cells stably expressing GFP-Rab7. Following homogenization using a ball-bearing cell cracker, a post nuclear supernatant (PNS) was prepared from these cells and loaded onto non-continuous Nycodenz-gradients, followed by centrifugation. **b**, Immunoblotting of HeLa cell lysates stably expressing GFP-Rab7 using a Rab7-specific antibody. **c**, Immunoblots of fractions enriched in early/recycling (EE/RE) and late (LE) endosomes (PNS, postnuclear supernatant, starting material), probed for marker proteins including EEA1 and Rab5 for early endosomes, Rab7 for late endosomes, and cathepsin D for late endosomes and lysosomes. **d**, Fluorescent images of the fractions from the Nycodenz gradient after particle deposition on glass coverslips. Early/recycling and late endosomes were concentrated at the boundary between 7.3% and 19%, and between 19% and 28% Nycodenz, respectively. Scale bar, 10 µm. **e**, Cartoon showing microinjection of endosomes expressing a TfnR-GFP in which GFP is fused to the luminal/extracellular domain of the receptor. If the endosomes are functional, they are expected to recycle to the plasma membrane, resulting in surface exposure of GFP. **f**, HeLa cells were microinjected with isolated TfnR-GFP containing endosomes. The cells were incubated at 37°C or 4°C for the indicated time period. They were then transferred into ice-cold medium containing anti-GFP antibody to block membrane traffic and to label surface-exposed GFP. After 60 min incubation, the cells were fixed and incubated with Cy3-conjugated anti-Rabbit antibody (red) and Alexa Fluor 633-Phalloidin (blue) to show the subcortical actin network. The panels on the right show a control with cells that were kept at 4°C through the whole experiment. Scale bar, 10 µm. **g**, Quantification of fluorescence intensity of the GFP signal on the cell surface 15 min after microinjection at 37°C and 4°C as described in (**f**). Error bars indicate SEM, $***P < 0.001$, determined by unpaired t test. **h**, GFP-labeling is restricted to the cell surface. XZ projection of cells whose surface was immunolabeled with anti-GFP antibody 15 min after microinjection of endosomes expressing TfnR-GFP. The cell surface is indicated by a dotted line in the upper panel. Scale bar, 10 µm.

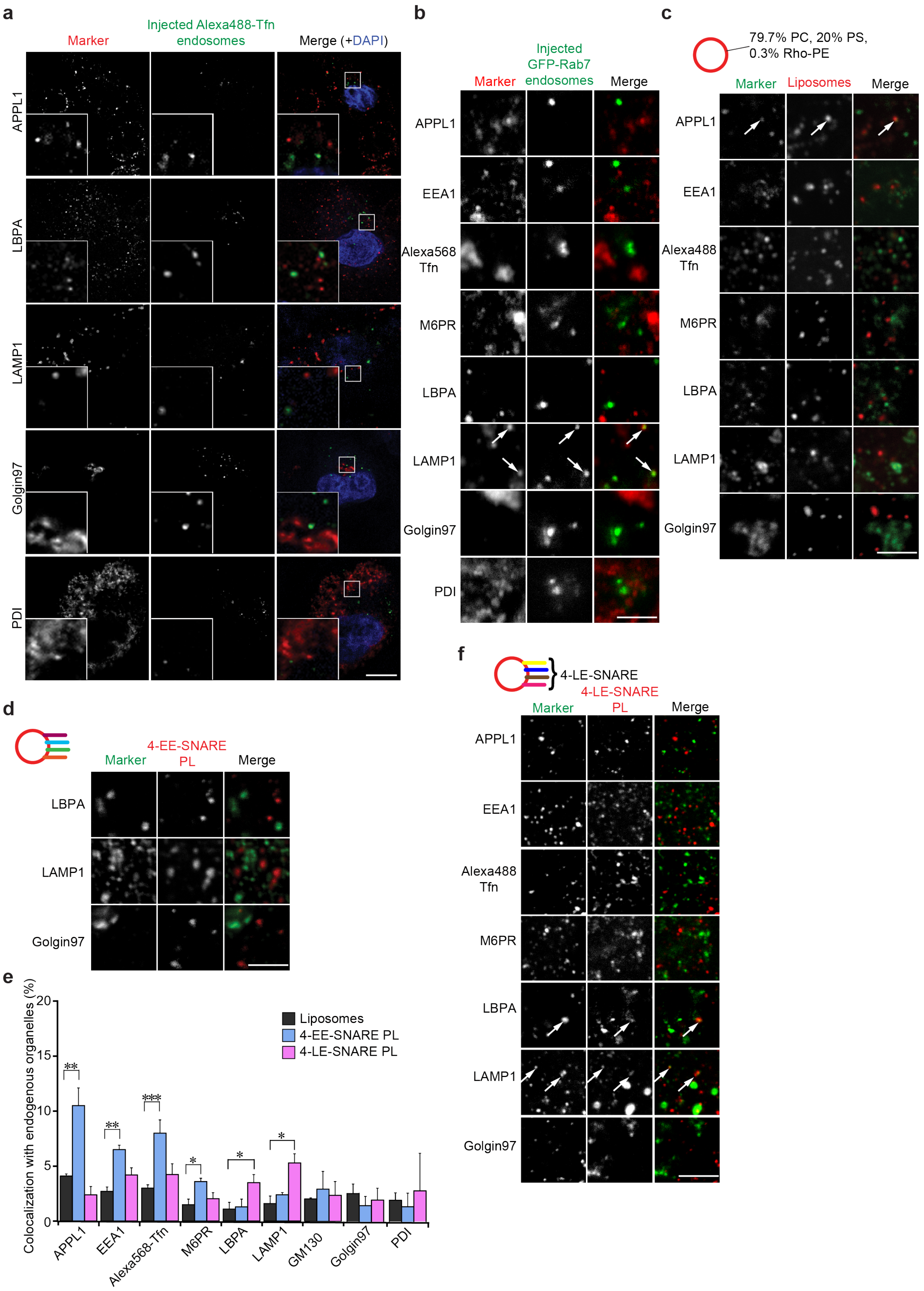

Supplementary Figure 2. Isolated endosomes and liposomes containing endosomal SNAREs

are targeted to their endogenous counterparts.

a, Representative confocal microscopy images of HeLa cells fixed 5 min after injection of endosomes containing internalized Alexa Fluor 488-Tfn. DAPI is an injection marker. Cells were immunolabeled for APPL1, LBPA, and LAMP1, Golgin97, PDI. Inserts show high magnifications of the boxed areas. Scale bar, 10 µm. **b**, Representative confocal microscopy images of HeLa cells fixed 5 min after injection of late endosomes labeled by GFP-Rab7 and DAPI. DAPI is an injection marker. Cells were immunolabeled for organelle markers. White arrows indicate co-localization between liposomes and organelle markers. Scale bar, 10 µm. **c** as in (**b**) but the cells were injected with protein-free liposomes. Scale bar, 2.5 µm. **d** as in(**b**) but the cells were injected with proteoliposomes (PLs) reconstituted with early endosomal SNAREs (4-EE-SNARE PL). Scale bar, 2.5 µm. **e**, Co-localization between injected liposomes containing either four early endosomal SNAREs (4-EE-SNARE PL, light gray), four late endosomal SNAREs (4-LE-SNARE PL, dark gray), or protein-free liposomes, respectively, and endogenous markers (in % of all labeled injected endosomes). Error bars indicate SEM, $^{*}P < 0.05$, $^{**}P < 0.01$, $^{***}P < 0.001$, determined by unpaired t test. **f** as in (**b**) but the cells were injected with proteoliposomes (PLs) reconstituted with late endosomal SNAREs (4-LE-SNARE PL). White arrows indicate co-localization between injected liposomes and organelle markers. Scale bar, 2.5 µm.

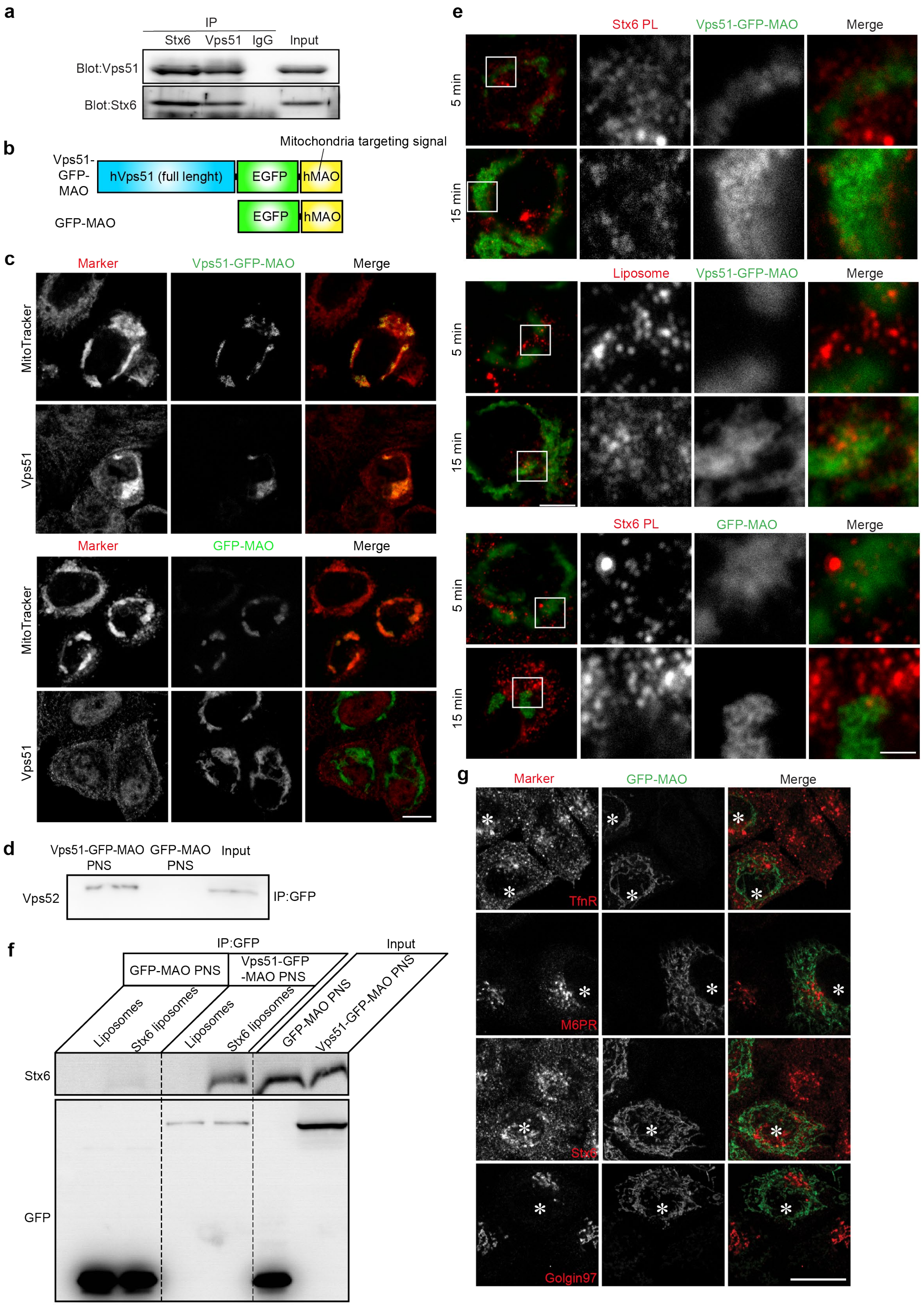
a
IP
Stx6
Vps51
IgG
Input
Blot:Vps51
Blot:Stx6
b
Mitochondria targeting signal
Vps51-GFP-MAO
hVps51 (full lenght)
EGFP
hMAO
GFP-MAO
c
Marker
Vps51-GFP-MAO
Merge
MitoTracker
Vps51
GFP-MAO
d
Vps51-GFP-MAO PNS
GFP-MAO PNS
Input
Vps52
IP:GFP
e
Stx6 PL
Liposome
5 min
15 min
f
IP:GFP
GFP-MAO PNS
Vps51-GFP -MAO PNS
Liposomes
Stx6 liposomes
Stx6
GFP
g
TfnR
M6PR
Golgin97

Supplementary Figure 3. Vps51 is necessary and sufficient for targeting Stx6 PL.

a, Immunoprecipitation of Vps51 and Stx6, respectively, results in the co-precipitation of the other protein. **b**, Constructs used for mitochondrial targeting. Vps51-GFP or GFP was fused to a fragment coding for the C-terminal region (residues 496-520) of monoamine oxidase-B (MAO) that was shown to be sufficient for localizing the protein to the outer mitochondria membrane. **c**, HeLa cells expressing Vps51-GFP-MAO or GFP-MAO were stained with MitoTracker or with an antibody specific for Vps51. Vps51-GFP-MAO or GFP-MAO co-localized with MitoTracker, indicating targeting of the fusion protein to the outer mitochondria membrane. Vps51-GFP-MAO, but not GFP-MAO, was recognized by the Vps51-specific antibody. Scale bar, 10 µm. **d**, Immunoprecipitation of Vps51-GFP-MAO or GFP-MAO from extracts of transfected cells with a GFP antibody. The immunoprecipitates were analyzed by immunoblotting for the presence of Vps52, another component of the GARP complex. **e**, Re-routing of Stx6 PL to the surface of mitochondria expressing Vps51-GFP. Note that the accumulation of Stx6 PL became evident 15 min after injection. No accumulation was observed in Vps51-GFP-MAO expressing cells injected liposomes or GFP-MAO expressing cells injected Stx 6 PL. Scale bar, 7.5 µm. **f**, In vitro interaction between mitochondria containing Vps51-GFP-MAO and proteoliposomes containing Stx6. Postnuclear supernatants from HeLa cells expressing GFP-MAO or Vps51-GFP-MAO were mixed with Stx6 liposomes and protein-free liposomes as control and then incubated for 30 min at 37°C. GFP-containing membranes were immunoisolated using GFP trap (see Methods) and probed by immunoblotting for GFP and Stx6. **g**, Expression of GFP-MAO does not change the distribution of endosomes or Golgi membranes. Scale bar, 10 µm.

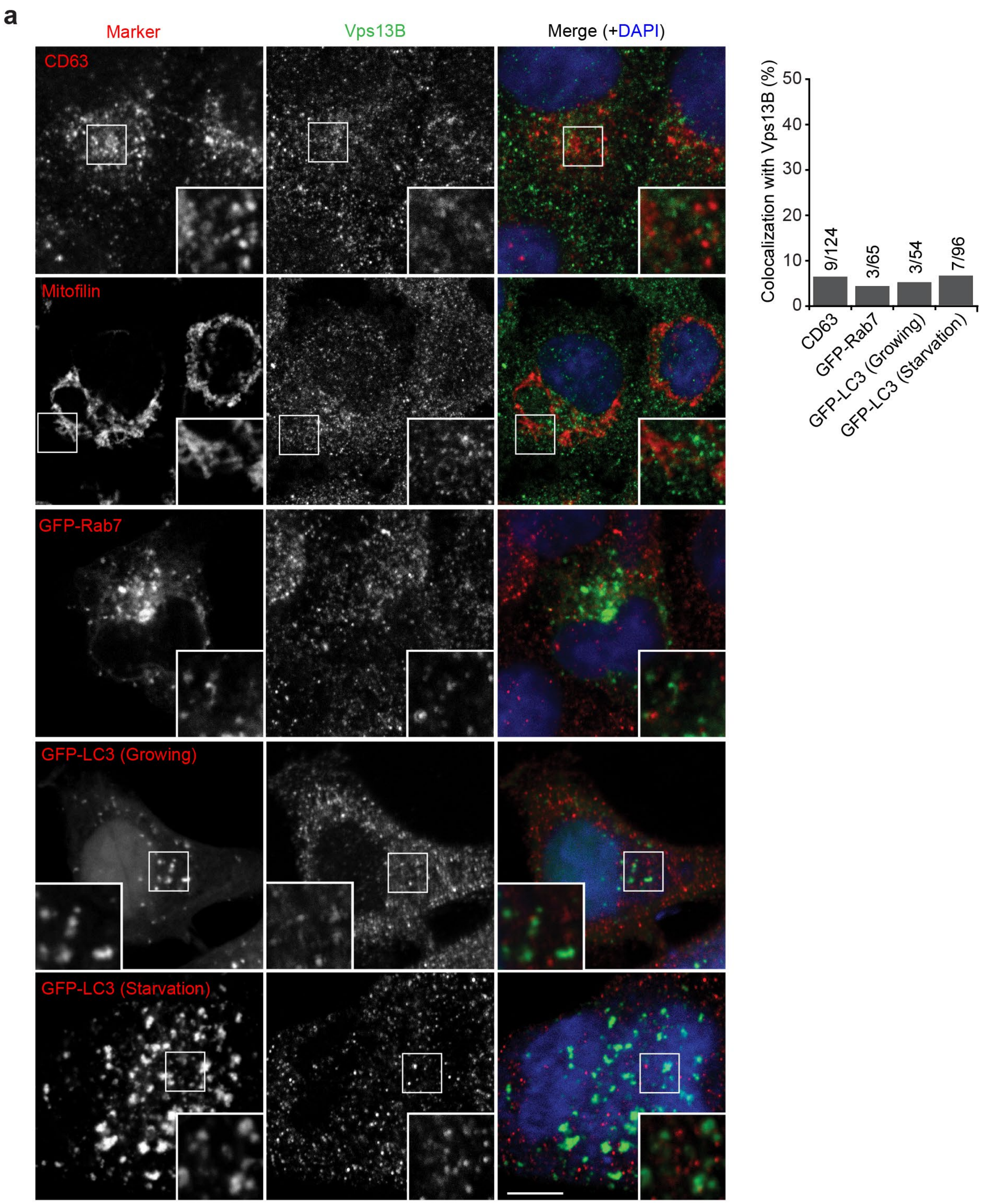

Supplementary Figure 4. Co-localization of endogenous Vps13B and organelle markers in HeLa cells.

a, Co-localization of endogenous Vps13B and various organelle markers in HeLa cells, detected by immunofluorescence using double-labeling for Vps13B and the proteins indicated by means of specific antibodies. A bar graph shows colocalization between organelle markers and Vps13B. Scale bar, 10 μm.

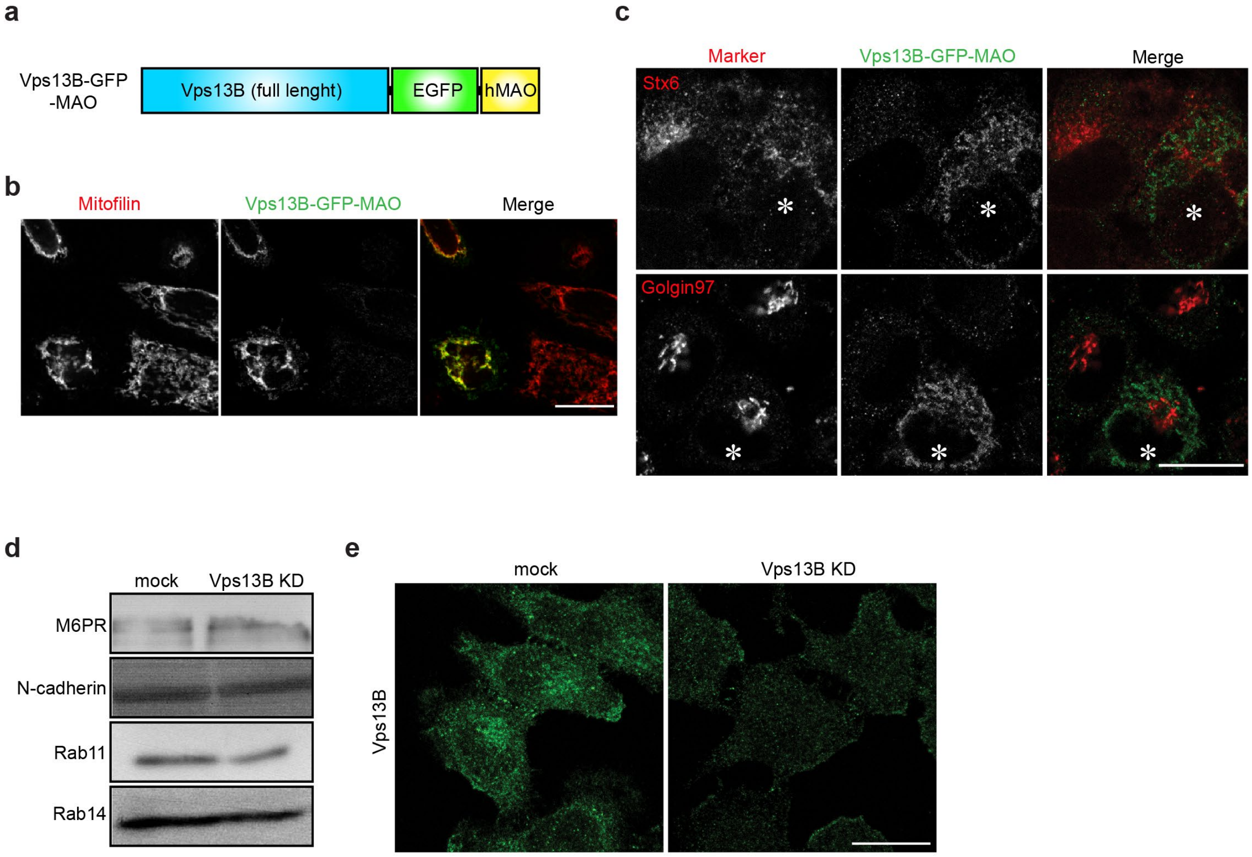

Supplementary Figure 5. Vps13B functions as a tethering protein.

a, Diagram showing the Vps13B construct used for mitochondrial targeting. Vps13B-GFP was fused to the C-terminal region (residues 496-520) of monoamine oxidase-B (MAO) that was shown to be sufficient for localizing the protein to the outer mitochondria membrane. **b**, HeLa cells expressing Vps13B-GFP-MAO were immunostained for mitofilin as mitochondrial marker and for GFP, showing co-localization. Scale bar, 20 µm. **c**, Change in the distribution of endogenous Stx6 upon expression of Vps13-GFP-MAO. Although there is no complete co-localization, the perinuclear accumulation of Stx6 seen in wild-type cells (probably recycling endosomes) was decreased. For comparison, the distribution of Golgin97 was not affected by expression of the construct. Asterisks mark Vps13B-GFP-MAO expressing cells. Scale bar, 20 µm. **d**, Knockdown of Vps13B does not change expression levels of M6PR, Rabs 11 and 14, or N-cadherin. Equal amounts of cell lysates were analyzed by immunoblotting using the respective antibodies. **e**, Immunofluorescence of mock and Vps13B KD cells with anti-Vps13B antibody.

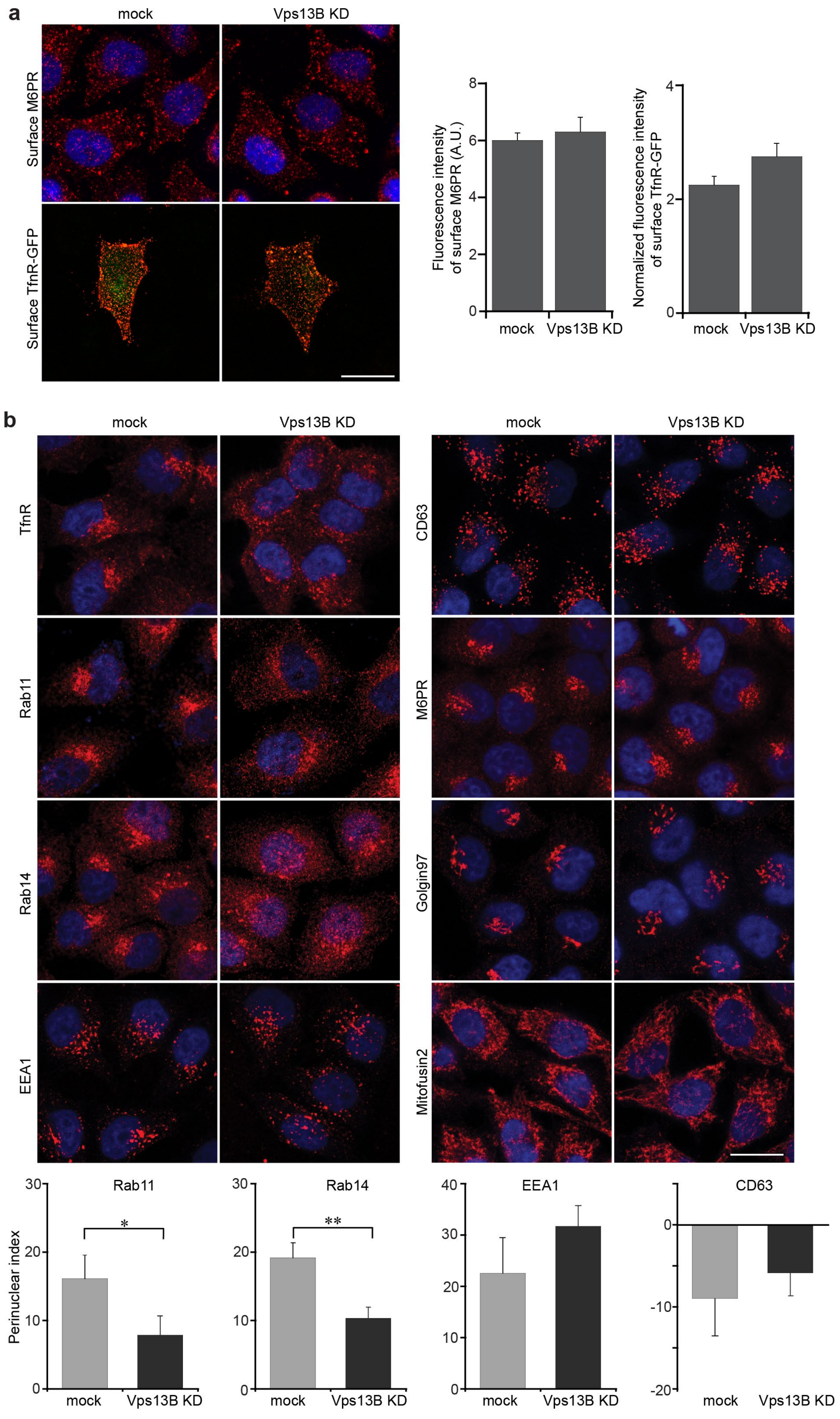

a
mock
Vps13B KD
Surface M6PR
Surface TfnR-GFP
Fluorescence intensity of surface M6PR (A.U.)
Normalized fluorescence intensity of surface TfnR-GFP
mock
Vps13B KD
b
mock
Vps13B KD
TfnR
Rab11
Rab14
EEA1
CD63
M6PR
Golgin97
Mitofusin2
Perinuclear index
Rab11
Rab14
EEA1
CD63
*
**
mock
Vps13B KD

Supplementary Figure 6. Phenotype analysis of Vps13B knock-down cells.

a, Surface exposure of M6PR and TfnR remains unchanged upon Vps13B knockdown. Quantification of fluorescence intensity is shown in the right side. **b**, The distribution of various vesicle markers was compared between wild-type and Vps13B KD cells. Note that the TGN (Golgin97 staining) was fragmented and the perinuclear accumulation of TfnR, Rab11 and Rab14 was significantly diminished in KD cells. Scale bar, 20 µm. Error bars indicate SEM, *P < 0.05, **P < 0.01, determined by unpaired t test.

Supplementary Table 1, Proteomic analysis of proteins binding to Stx6/Stx13 liposomes.

	Accession No.	Gene name	Protein name	Fold change
1	Q13347	EIF3I	Eukaryotic translation initiation factor 3 subunit I	10.00
2	Q15477	SKIV2L	Helicase SKI2W	8.00
3	E9PB90	HK2	Hexokinase-2	8.00
4	P04049	RAF1	RAF proto-oncogene serine/threonine-protein kinase	8.00
5	Q7Z7G8-2	VPS13B	Vacuolar protein sorting-associated protein 13B	7.00
6	Q5VT52-2	RPRD2	Regulation of nuclear pre-mRNA domain-containing protein 2	6.50
7	O15160-2	POLR1C	DNA-directed RNA polymerases I and III subunit RPAC1	6.00
8	O15357	INPPL1	Phosphatidylinositol 3,4,5-trisphosphate 5-phosphatase 2	6.00
9	Q13535-2	ATR	Serine/threonine-protein kinase ATR	6.00
10	Q05932-3	FPGS	Folylpolyglutamate synthase, mitochondrial	6.00

The 10 proteins most highly enriched on Stx6/Stx13 liposomes compared to Stx6 liposomes. Stx6/Stx13 or Stx6 liposomes binding proteins were identified by proteomics (MS-MS) and analyzed with a Scaffold software. Fold change values were calculated from 2 independent experiments.

Supplementary Table 2, Primer list

Cloning of full length human Vps51 into pEGFP-N vectors

Name	Sequence
EcoR-VPS51 Fwd	5'-TCG AAT TCG GCA CGA GGG TTG GAA CGA TG-3'
VPS51-BamHI Rev	5'-GTG GAT CCT GCC GCG CTC GCA GAT GAC-3'

Cloning of full length mouse Vps13B into pEGFP vectors

Name	Sequence
mouse Vps13B Fragment 1 Fwd	5'-CGA GCT CAA GCT TCG AAT TCA CCA TGC TGG AGT CGT ACG TAA CTC-3'
mouse Vps13B Fragment 1 Rev	5'-GAT TGA GCC CTC AAG AGT TG-3'
mouse Vps13B Fragment 2 Fwd	5'-CAA CTC TTG AGG GCT CAA TC-3'
mouse Vps13B Fragment 2 Rev	5'-CTG TAC ATG TGT AGT CAG AG-3'
mouse Vps13B Fragment 3 Fwd	5'-CTC TGA CTA CAC ATG TAC AG-3'
mouse Vps13B Fragment 3 Rev	5'-CAG TGT CAC TAC CTC ACC ATT C-3'
mouse Vps13B Fragment 4 Fwd	5'-GAA TGG TGA GGT AGT GAC ACT G-3'
mouse Vps13B Fragment 4 Rev	5'-CCG GTG GAT CCC GGG CCC GCG GGG AGA AGCCTT TCC TCA GGG-3'

Cloning of the mitochondria targeting signal of MAO into pEGFP-N vector

Name	Sequence
BsrGI-MAO fwd	5'-AGC TGT ACA AGT CTG TGG ATG TCC CTG CAC-3'
MAO-NotI	5'-AAT GCG GCC GCT TAG ACT CTC ACA AGT AGC-3'
