# Supplementary material for: SNAREs define targeting specificity of trafficking vesicles by combinatorial interaction with tethering factors
Source: Nat Commun. 2019 Apr 8;10:1608. doi: 10.1038/s41467-019-09617-9 (PMC6453939; doi:10.1038/s41467-019-09617-9)
Supplement: Supplementary file 3 — Reporting Summary Checklist [file 41467_2019_9617_MOESM3_ESM.pdf]

nature research

Corresponding author(s): NCOMMS-18-32535B

Reporting Summary

Nature Research wishes to improve the reproducibility of the work that we publish. This form provides structure for consistency and transparency in reporting. For further information on Nature Research policies, see Authors & Referees and the Editorial Policy Checklist.

Statistical parameters

When statistical analyses are reported, confirm that the following items are present in the relevant location (e.g. figure legend, table legend, main text, or Methods section).

n/a	Confirmed	
☐	☒	The exact sample size (n) for each experimental group/condition, given as a discrete number and unit of measurement
☐	☒	An indication of whether measurements were taken from distinct samples or whether the same sample was measured repeatedly
☐	☒	The statistical test(s) used AND whether they are one- or two-sided<br>*Only common tests should be described solely by name; describe more complex techniques in the Methods section.*
☒	☐	A description of all covariates tested
☐	☒	A description of any assumptions or corrections, such as tests of normality and adjustment for multiple comparisons
☐	☒	A full description of the statistics including central tendency (e.g. means) or other basic estimates (e.g. regression coefficient) AND variation (e.g. standard deviation) or associated estimates of uncertainty (e.g. confidence intervals)
☒	☐	For null hypothesis testing, the test statistic (e.g. F, t, r) with confidence intervals, effect sizes, degrees of freedom and P value noted<br>*Give P values as exact values whenever suitable.*
☒	☐	For Bayesian analysis, information on the choice of priors and Markov chain Monte Carlo settings
☒	☐	For hierarchical and complex designs, identification of the appropriate level for tests and full reporting of outcomes
☒	☐	Estimates of effect sizes (e.g. Cohen's d, Pearson's r), indicating how they were calculated
☐	☒	Clearly defined error bars<br>*State explicitly what error bars represent (e.g. SD, SE, CI)*

Our web collection on statistics for biologists may be useful.

Software and code

Policy information about availability of computer code

Data collection	All the images with microscopy were collected with Zen software (Carl Zeiss)<br>Western blot images were collected with LAS-1000 (FujiFilm)
Data analysis	Colocalization between injected liposomes and endogenous organelles were analyzed with a Matlab algorithm provided by Prof. Silvio Rizzoli. Fluorescence intensity and perinuclear index was analyzed with Image J software.

For manuscripts utilizing custom algorithms or software that are central to the research but not yet described in published literature, software must be made available to editors/reviewers upon request. We strongly encourage code deposition in a community repository (e.g. GitHub). See the Nature Research guidelines for submitting code & software for further information.

Data

Policy information about availability of data

All manuscripts must include a data availability statement. This statement should provide the following information, where applicable:

- Accession codes, unique identifiers, or web links for publicly available datasets
- A list of figures that have associated raw data
- A description of any restrictions on data availability

The plasmids and experimentally data that support the findings of this study are available from the corresponding authors upon reasonable request.

Field-specific reporting

Please select the best fit for your research. If you are not sure, read the appropriate sections before making your selection.

☒ Life sciences ☐ Behavioural & social sciences ☐ Ecological, evolutionary & environmental sciences

For a reference copy of the document with all sections, see nature.com/authors/policies/ReportingSummary-flat.pdf

Life sciences study design

All studies must disclose on these points even when the disclosure is negative.

Sample size	The number of independent experiments used for the calculation of mean values and S.E.M. values is given in the legend to each panel. No statistical methods were applied to predetermine sample size.
Data exclusions	No data were excluded.
Replication	Experiments were repeated at least three independent experiments. If there were outliers, experiments were performed more to reduce S.E.M..
Randomization	no applicable
Blinding	Not applied in this study

Reporting for specific materials, systems and methods

Materials & experimental systems

n/a	Involved in the study
☒	☐ Unique biological materials
☐	☒ Antibodies
☐	☒ Eukaryotic cell lines
☒	☐ Palaeontology
☒	☐ Animals and other organisms
☒	☐ Human research participants

Methods

n/a	Involved in the study
☒	☐ ChIP-seq
☒	☐ Flow cytometry
☒	☐ MRI-based neuroimaging

Antibodies

Antibodies used

Primary Antibodies
Anti-APPL1 (Rabbit monoclonal, #3858, Cell Signaling Technology)
anti LC3B (Rabbit monoclonal, #4599, Cell Signaling Technology)
anti-Rab7 (Rabbit monoclonal, #9367, Cell Signaling Technology)
anti-EEA1 (Mouse monoclonal, clone 14, #612006, Becton, Dickinson)
anti-GM130 (Mouse monoclonal, clone 35, #560257, Becton, Dickinson)
anti-M6PR (Mouse monoclonal, clone 2G11, ab2733, Abcam)
anti-LAMP1 (Rabbit polyclonal, ab24170, Abcam)
anti-mitofilin (Mouse monoclonal, clone 2E4AD5, ab110329, Abcam)
anti-mitofusion2 (Rabbit polyclonal, ab101055, Abcam)
anti-cathepsin D (Rabbit polyclonal, ab6313, Abcam)
anti-transferrin receptor (Mouse monoclonal, clone H68.4, sc-65882, Santa Cruz Biotechnology)

anti-LBPA (Mouse monoclonal, clone 6C4, Z-PLBPA, Echelon)
anti-Golgin97 (Mouse monoclonal, clone CDF4, A-21270, Thermo Fisher Scientific)
anti-RFP (Rabbit polyclonal, R10367, Thermo Fisher Scientific)
anti-Vps52 (Rabbit polyclonal, PA5-24408, Thermo Fisher Scientific)
anti-Rab11 (Rabbit polyclonal, 71-5300, Thermo Fisher Scientific)
anti-PDI (Mouse monoclonal, clone 7H25L2, 700782, ABfinity)
anti-Vps51 (Rabbit polyclonal, HPA061447, Atlas antibodies)
anti-Vps13B (Rabbit polyclonal, HPA043865, Atlas antibodies)
anti-CD63 (Mouse monoclonal, clone H5C6, Developmental Studies Hybridoma Bank)
anti-Rab6 (Rabbit polyclonal, 10187-2-P,Proteintech)
anti-Rab35 (Rabbit polyclonal,11329-2-AP ,Proteintech)
anti-Rab14 (Rabbit polyclonal, R0656, Sigma-Aldrich)
anti-GFP (Rabbit polyclonal, 132002, Synaptic system)
anti-α-tubulin (Mouse Monoclonal, clone 3A2, 302211, Synaptic system)
anti-beta-actin (Rabbit polyclonal, 251003, Synaptic system)
anti-Rab5 (Mouse Monoclonal, clone 621.3, 108011, Synaptic system)
anti-syntaxin 6 (Rabbit polyclonal, 110062, Synaptic system)
anti-syntaxin 13 (Rabbit polyclonal, 110132, Synaptic system)
anti-syntaxin 4 (Rabbit polyclonal, 110042, Synaptic system)
anti-syntaxin 16 (Rabbit polyclonal, 110162, Synaptic system)
anti-VAMP4 (Rabbit polyclonal, 136002, Synaptic system)
Secondary antibodies
Alexa Fluor 488-conjugated goat anti-mouse (115-545-166, Jackson ImmunoResarch Laboratories)
Cy3-conjugated goat anti-mouse (115-165-146, Jackson ImmunoResarch Laboratories)
Cy5-conjugated goat anti-mouse (115-175-166, Jackson ImmunoResarch Laboratories)
HRP-conjugated goat anti-mouse (STAR117P, BioRad)
Alexa Fluor 488-conjugated goat anti-rabbit (111-545-144, Jackson ImmunoResarch Laboratories)
Cy3-conjugated goat anti-rabbit (111-165-144, Jackson ImmunoResarch Laboratories)
Cy5-conjugated goat anti-rabbit (111-175-144, Jackson ImmunoResarch Laboratories)
HRP-conjugated goat anti-rabbit (5196-2504, BioRad)

Validation

Anti-APPL1 (Rabbit polyclonal, #3858, Cell Signaling Technology,https://www.cstj.co.jp/search/index.php?queryString=appl1&x=0&y=0)
anti LC3B (#4599, Cell Signaling Technology, https://www.cstj.co.jp/products/4599.html)
anti-Rab7 (#9367, Cell Signaling Technology, https://www.cstj.co.jp/products/9367.html)
anti-EEA1 (#612006, Becton, Dickinson, https://www5.bdj.co.jp/reagents/view/610456)
anti-GM130 (#560257, Becton, Dickinson, https://www5.bdj.co.jp/reagents/view/610822)
anti-M6PR (ab2733, Abcam, https://www.abcam.co.jp/m6pr-cation-independent-antibody-2g11-ab2733.html)
anti-LAMP1 (ab24170, Abcam, https://www.abcam.co.jp/lamp1-antibody-lysosome-marker-ab24170.html)
anti-mitofilin (ab110329, Abcam, https://www.abcam.co.jp/mitofilin-antibody-2e4ad5-mitochondrial-marker-ab110329.html)
anti-mitofusion2 (ab101055, Abcam, https://www.abcam.co.jp/mitofusin-2-antibody-ab101055.html)
anti-cathepsin D (ab6313, Abcam, https://www.abcam.co.jp/cathepsin-d-antibody-ctd-19-ab6313.html)
anti-transferrin receptor (sc-65882, Santa Cruz Biotechnology, https://www.scbt.com/scbt/ja/product/cd71-antibody-h68-4?requestFrom=search)
anti-LBPA (Z-PLBPA, Echelon, http://www.echelon-inc.com/index.php?module=Products&func=detail&id=767)
anti-Golgin97 (A-21270, Thermo Fisher Scientific, https://www.thermofisher.com/antibody/product/Golgin-97-Antibody-clone-CDF4-Monoclonal/A-21270)
anti-RFP (Rabbit polyclonal, R10367, Thermo Fisher Scientific, https://www.thermofisher.com/antibody/product/RFP-Tag-Antibody-Polyclonal/R10367)
anti-Vps52 (PA5-24408, Thermo Fisher Scientific, https://www.thermofisher.com/antibody/product/VPS52-Antibody-Polyclonal/PA5-24408)
anti-Rab11 (Rabbit polyclonal, 71-5300, Thermo Fisher Scientific, https://www.thermofisher.com/antibody/primary/target/rab11)
anti-PDI (700782, ABfinity, https://www.thermofisher.com/antibody/product/PDIA2-Antibody-clone-7H25L2-Monoclonal/700782)
anti-Vps51 (HPA061447, Atlas antibodies, https://atlasantibodies.com/products/VPS51-antibody-HPA061447)
anti-Vps13B (HPA043865, Atlas antibodies, https://atlasantibodies.com/products/VPS13B-antibody-HPA043865)
anti-CD63 (Mouse monoclonal, clone H5C6, Developmental Studies Hybridoma Bank, http://dshb.biology.uiowa.edu/H5C6)
anti-Rab6 (10187-2-P,Proteintech, https://www.ptglab.com/products/RAB6A-Antibody-10187-2-AP.htm)
anti-Rab35 (11329-2-AP ,Proteintech, https://www.ptglab.com/Products/RAB35-Antibody-11329-2-AP.htm)
anti-Rab14 (R0656, Sigma-Aldrich, https://www.sigmaaldrich.com/catalog/product/sigma/r0656?lang=de&region=DE)
anti-GFP (132002, Synaptic system, https://www.sysy.com/products/gfp/facts-132002.php)
anti-α-tubulin (clone 3A2, 302211, Synaptic system, https://www.sysy.com/products/tubulin/facts-302211.php)
anti-beta-actin (Rabbit polyclonal, 251003, Synaptic system, https://www.sysy.com/products/b-actin/facts-251003.php)
anti-Rab5 (108011, Synaptic system, https://www.sysy.com/products/rab5/facts-108011.php)
anti-syntaxin 6 (110062, Synaptic system, https://www.sysy.com/products/syntaxin6/facts-110062.php)
anti-syntaxin 13 (110132, Synaptic system, https://www.sysy.com/products/syntaxin13/facts-110132.php)
anti-syntaxin 4 (110042, Synaptic system, https://www.sysy.com/products/syntaxin4/facts-110042.php)
anti-syntaxin 16 (110162, Synaptic system, https://www.sysy.com/products/syntaxin16/facts-110162.php)
anti-VAMP4 (136002, Synaptic system, https://www.sysy.com/products/vamp4/facts-136002.php)

Eukaryotic cell lines

Policy information about cell lines

Cell line source(s)	HeLa cell, Vero cell
Authentication	none
Mycoplasma contamination	The cell lines were not tested for Mycoplasma contamination
Commonly misidentified lines (See ICLAC register)	none
